# Tumour-infiltrating lymphocytes do not predict survival in human papillomavirus-associated oropharyngeal squamous cell carcinoma: a single institution retrospective review

**DOI:** 10.1017/S0022215126104538

**Published:** 2026-05

**Authors:** Michael Fitzsimons, Grace O'Flanagan, Shane Brennan, Hannah O'Driscoll, Susan Kennedy, Conrad Timon, Robbie Seton Rowan Woods

**Affiliations:** 1 Royal College of Surgeons in Ireland, Ireland; 2 Royal Victoria Eye and Ear Hospital, Dublin, Ireland; 3 St. James’ Hospital, Dublin, Ireland; 4 St. Luke’s Radiation Oncology Network, Dublin, Ireland; 5 Department of Otolaryngology, Beaumont Hospital, Dublin, Ireland

**Keywords:** oropharyngeal neoplasms, lymphocytes, tumor-infiltrating, papillomavirus infections, prognosis

## Abstract

**Objective:**

Human papillomavirus (HPV) associated oropharyngeal squamous cell carcinoma (OPSCC) exhibits a favourable prognosis compared to non-HPV OPCSCC. Tumour infiltrating lymphocytes (TILs) may predict better survival outcomes for patients with HPV+OPSCC.

**Methods:**

A retrospective review of patients treated for HPV+OPSCC between 2003-2018. TIL density scores for each patient’s tumour specimen were determined. Patients were categorised as TIL-Hi or TIL-Lo. Survival outcomes for the two TIL groups were compared.

**Results:**

Fifty-seven patients, 33 in the TIL-Hi group, were included. 5-year overall survival was 81.8% in the TIL-Hi and 75% in the TIL-Lo groups. 5-year disease specific survival was 87.9% and 87.5%, respectively. There was no statistically significant difference in survival outcomes between TIL-Hi and TIL-Lo.

**Conclusion:**

TIL density in HPV+OPSCC has been reported to predict survival outcomes. This is not supported by the findings of this study. Further prospective evaluation with a standardised scoring technique may fully elicit the prognostic value of TILs in HPV+OPSCC.

## Introduction

Oropharyngeal squamous cell carcinoma (SCC) associated with human papillomavirus (HPV) carries a favourable prognosis when compared with non-HPV oropharyngeal SCC. It is well established that oropharyngeal SCC associated with HPV exhibits greater treatment response and survival versus non-HPV oropharyngeal SCC.^1^^–^^3^ This has been reflected in the most recent (eighth) edition of the American Joint Committee on Cancer cancer staging manual^4^^,^^5^ wherein oropharyngeal SCC associated with HPV has a distinct tumour–node–metastasis (TNM) classification. With that in mind, there exists potential for less intense treatment regimens for oropharyngeal SCC associated with HPV that have the potential to reduce morbidity while preserving efficacy.

A subgroup of patients with oropharyngeal SCC associated with HPV still has a poor prognosis, with up to 36 per cent of patients experiencing recurrence within 8 years^6^ and up to 19 per cent not surviving beyond 3 years.^1^ Attempts to substitute concurrent cisplatin chemotherapy with cetuximab, an epidermal growth factor receptor (EGFR) inhibitor, in patients treated with primary chemoradiotherapy demonstrated no benefit in terms of toxicity reduction and in fact was detrimental to overall survival and tumour control.^7^^,^^8^ Hence, there is a need to identify predictors of a positive prognosis for patients with oropharyngeal SCC associated with HPV to enable appropriate selection for de-intensified treatment protocols and ensure optimal outcomes.^9^

Tumour-infiltrating T-lymphocytes specific to HPV within the tumour microenvironment may infer a greater immune response, improved treatment response and favourable survival outcomes in patients with oropharyngeal SCC associated with HPV.^10^ Several studies have identified a positive relationship between the density of tumour-infiltrating T-lymphocytes in the tumour microenvironment and patient outcomes in oropharyngeal SCC associated with HPV.^11^^–^^13^ Further validation of the prognostic role of tumour-infiltrating T-lymphocytes in oropharyngeal SCC associated with HPV is needed if it is to be routinely utilised in treatment decisions. To that end, we retrospectively investigated the prognostic value of tumour-infiltrating T-lymphocytes for patients diagnosed with oropharyngeal SCC associated with HPV in a single institution in Dublin, Ireland.

## Methods

A list of patients diagnosed with oropharyngeal SCC associated with HPV in the Royal Victoria Eye and Ear Hospital, Dublin between 2003 and 2018 was retrospectively compiled. This study was approved by the Hospital Research Ethics Committee and is in compliance with the Helsinki Declaration. Human papillomavirus status was determined at the time of diagnosis with p16 overexpression of more than 70 per cent used as a surrogate for HPV positivity. In some cases, DNA in situ hybridisation or polymerase chain reaction had already been carried out and so this information was included. A retrospective chart review was undertaken and a database populated from clinical notes, histopathology and radiology reports. Clinical data including age, sex, smoking status and pack year history, TNM stage, recurrence, survival and follow-up duration (to a maximum of five years) were extracted from the records. Patients who declined conventional treatment or who were treated with palliative intent were excluded from the study.

### Tumour-infiltrating T-lymphocyte scoring

For each patient, tumour-infiltrating lymphocyte density was scored by two pathologists (SK, SB) using archived whole-tumour haematoxylin and eosin-stained slides. Each haematoxylin and eosin slide was assessed and reported independently by each examiner. The scoring algorithm utilised was described by Marsh *et al*.^14^ and utilised in subsequent studies.^12^^,^^13^ The characteristics of tumour-infiltrating T-lymphocytes include that they are located at the invasive tumour front. Lymphocytes counted include those located between isolated single tumour cells or between islands and/or nests of tumour cells. In addition, as squamous cells are characteristically cohesive, lymphocytes within islands and/or nests of tumour cells were included. Scoring was conducted on a 10× magnification and 2.5 objective lens on the same microscope (Nikon Eclipse 50i, Tokyo, Japan).

Tumour-infiltrating T-lymphocytes were categorised into two groups: high and low. High tumour-infiltrating T-lymphocytes was defined as having a diffuse infiltrate present in more than 20 per cent of tumour and stroma. Low tumour-infiltrating T-lymphocytes was defined as having a weak or absent infiltrate present in less than 20 per cent of tumour and stroma. Cases with discrepancies were reviewed together by both pathologists and an agreed score was provided before submitting to statistical analysis.

### Statistical analysis

Patients were grouped according to tumour-infiltrating T-lymphocyte score (high or low). Clinical characteristics between the two groups were compared using chi-square, Fisher’s exact test and *t*-test, where appropriate.

Survival analysis was conducted to compare five-year overall survival, disease-free survival and disease-specific survival between the two groups. Kaplan–Meier survival curves were plotted for disease-free survival, disease-specific survival and overall survival. Log-rank test was employed to compare survival functions. Statistical significance was considered as *p* less than 0.05. Statistical analysis was performed using Stata/SE 16.0 for Mac (StataCorp, Texas, USA).

## Results

Seventy-four patients diagnosed with HPV-positive oropharyngeal SCC (oropharyngeal SCC associated with HPV) during the study period of July 2003 to December 2018 were identified. There was insufficient remaining archived tissue sample for tumour-infiltrating T-lymphocyte analysis for 15 patients, and 2 patients were treated with palliative intent (1 due to metastatic disease at presentation and 1 due to co-morbidities). Thus 57 patients were included in the final review. HPV DNA polymerase chain reaction and/or in situ hybridisation was performed on 27 of 57 cases which confirmed HPV positivity.

The mean age at diagnosis was 58 years (standard deviation, 8.6 years) with a male-to-female ratio of 5.3:1. The median follow-up duration was 60 months (interquartile range, 57–60 months). There were 12 deaths observed in the 5-year follow-up period. For the remaining 45 patients, complete 5-year follow up was available for 42 (i.e. complete 5-year survival data were recorded for 54 of 57 patients (94.7 per cent).

There were 33 of 57 patients (57.9 per cent) in the high tumour-infiltrating T-lymphocyte group. There was no statistically significant difference between the high and low tumour-infiltrating T-lymphocytes groups with regards to gender, follow up, smoking history, T stage, N stage or overall stage (Table 1). There was a trend towards older age in the low tumour-infiltrating T-lymphocytes group, but this was not statistically significant (*p* = 0.12).

**Table 1.** Overview characteristics Table 1 long description.

	High tumour-infiltrating T-lymphocytes (*n* = 33)	Low tumour-infiltrating T-lymphocytes (*n* = 24)	*p* value
Age (mean ± SD; years)	56.5 ± 8.8	60.1 ± 8.1	*0.12*
Sex (*n* (%))			*0.47*
– Female	4 (12.1)	5 (20.8)	
– Male	29 (87.9)	19 (79.2)	
Follow up (median (IQR); months)	60 (57−60)	60 (43.5−60)	
Smoking history (*n* (%))			*0.61*
– Less than 10 pack-years	16 (48.5)	10 (41.7)	
– Greater than 10 pack-years	17 (51.5)	14 (58.3)	
T stage (*n* (%))			*0.17*
– T1	7 (21.2)	3 (12.5)	
– T2	13 (39.4)	8 (33.3)	
– T3	10 (30.3)	5 (20.8)	
– T4	3 (9.1)	8 (33.3)	
N stage (*n* (%))			*0.57*
– N0	5 (15.1)	7 (29.2)	
– N1	20 (60.7)	14 (58.3)	
– N2	5 (15.1)	2 (8.3)	
– N3	3 (9.1)	1 (4.2)	
Stage (*n* (%))			*0.26*
– I	16 (48.5)	9 (37.5)	
– II	11 (33.3)	6 (25.0)	
– III	6 (18.2)	9 (37.5)	

SD = standard deviation; IQR = interquartile range.

There were 6 deaths (6 of 33, 18.2 per cent) observed in the high tumour-infiltrating T-lymphocytes group during 5-year follow up, of which 4 (4 of 33, 12.1 per cent) were due to oropharyngeal SCC. Six deaths (6 of 24, 25 per cent) occurred in the low tumour-infiltrating T-lymphocytes group. Three (3 od 24, 12.5 per cent) deaths in the low tumour-infiltrating T-lymphocytes group were due to oropharyngeal SCC. There was no statistically significant difference in 5-year overall survival (logrank, *p* = 0.46) or 5-year disease-specific survival (logrank, *p* = 0.88) between the two tumour-infiltrating T-lymphocyte groups. Disease-free survival at 5 years was similar between the two groups (logrank, *p* = 0.73).

## Discussion

It is well established that oropharyngeal SCC associated with HPV exhibits better survival outcomes when compared with non-HPV oropharyngeal SCC.^1^^–^^3^ While this raises the prospect of less intense treatment regimens for oropharyngeal SCC associated with HPV, it is important to remain mindful of the fact that a small subgroup within the oropharyngeal SCC associated with HPV cohort still has a poor prognosis. As such, a greater understanding of prognostic indicators in oropharyngeal SCC associated with HPV is required.

Several studies have reported the prognostic value of tumour-infiltrating lymphocytes in oropharyngeal SCC associated with HPV, with a higher density of tumour-infiltrating T-lymphocyte on haematoxylin and eosin-stained sections correlated with improved survival.^12^^,^^13^^,^^15^^–^^19^ A recent meta-analysis of 11 studies found tumour-infiltrating T-lymphocytes in oropharyngeal SCC to be a reliable prognostic indicator with improved disease-free, disease-specific and overall survival (hazard ration (HR) = 0.39, HR = 0.32, HR = 0.38).^20^ It was concluded that assessment of tumour-infiltrating T-lymphocytes could become part of routine practice ‘for better risk stratification and treatment planning in oropharyngeal SCC’.

**Figure 1.**
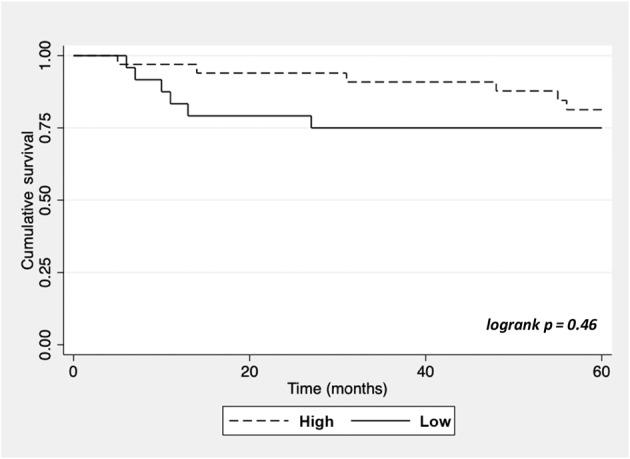
Five-year overall survival. Figure 1 long description.

**Figure 2.**
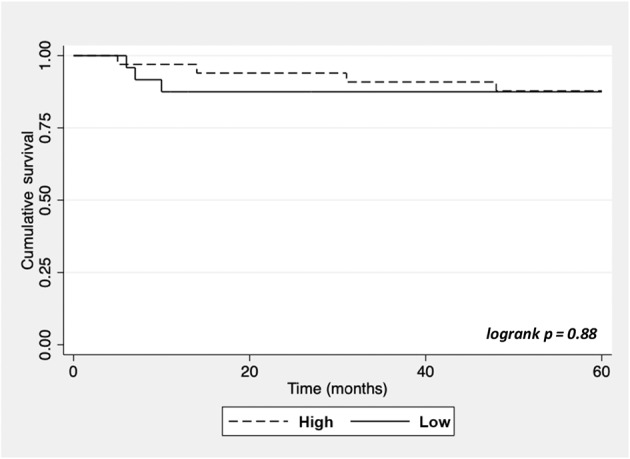
Five-year disease-specific survival. Figure 2 long description.

**Figure 3.**
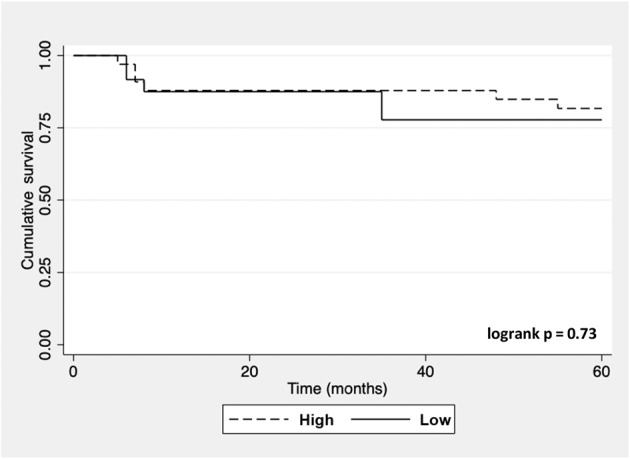
Five-year disease-free survival. Figure 3 long description.

The findings from our study do not support the utility of tumour-infiltrating T-lymphocytes alone as a prognostic marker in oropharyngeal SCC associated with HPV. We found similar outcomes for high and low tumour-infiltrating T-lymphocytes groups in terms of 5-year overall survival (*p* = 0.46) (Figure 1), 5-year disease-specific survival (*p* = 0.88) (Figure 2) and 5-year disease-free survival (*p* = 0.73) (Figure 3), although there was a slight trend towards improved survival in the high tumour-infiltrating T-lymphocytes group.

There are a number of reasons as to why the observed outcomes in our review may be in contrast to the findings of other published studies. These include the small sample size, retrospective study design and use of archived tissue samples, lack of lymphocyte subtype analysis and interobserver variability in tumour-infiltrating T-lymphocyte scoring. Nonetheless, the published literature on this topic to date is based on similar study designs. Several studies include both oropharyngeal SCC associated with HPV and non-HPV oropharyngeal SCC cases in their analysis. This study benefits from solely focusing on oropharyngeal SCC associated with HPV as this is an entity distinct from non-HPV oropharyngeal SCC.

The 5-year overall survival for oropharyngeal SCC associated with HPV is typically greater than 80 per cent.^1^^,^^21^^,^^22^ Overall 5-year survival was 79 per cent in our cohort. This comprised 5-year overall survival of 81.8 and 75 per cent for the high and low tumour-infiltrating T-lymphocytes groups, respectively. Ruangritchankul *et al*.^12^ (*n* = 232) reported 5-year overall survival greater than 80 per cent for all patients, which comprised approximately 88 and 46 per cent 5-year overall survival for the high and low tumour-infiltrating T-lymphocytes groups, respectively. Disease-specific survival at 3 years for 148 cases of oropharyngeal SCC associated with HPV reported by Ward *et al*.^13^ was 96, 76 and 59 per cent for high, moderate and low tumour-infiltrating T-lymphocytes. By comparison, 3-year disease-specific survival in our study was 90.9 per cent for the high tumour-infiltrating T-lymphocytes group and 87.5 per cent for the low tumour-infiltrating T-lymphocytes group. This demonstrates that the observed mortality rate in our low tumour-infiltrating T-lymphocytes cohort was lower than expected compared with rates reported in the literature.

The utility of using tumour-infiltrating T-lymphocyte density to stratify survival outcomes in oropharyngeal SCC associated with HPV lies in using a simple, reproducible, inexpensive test. Hence, several authors,^12^^,^^15^^,^^17^ including ourselves, have examined tumour-infiltrating T-lymphocyte scoring performed on haematoxylin and eosin staining without investigating lymphocyte subtype analysis. That said, T-cell subtype analysis has identified that, for example, CD8^13,^^,^^23^ and CD3^24^^,^^25^ cells in the tumour and stroma predict better survival. Conversely, the density of FOXP3, related to regulatory T-cells, can predict better survival in some cancers, while indicating worse prognosis in others.^26^

The value and prognostic direction of FOXP3 in head and neck squamous cell carcinoma (HNSCC) is also subject to conflicting reports.^24^^,^^25^^,^^27^^–^^30^ Acknowledging that the proportion of FOXP3 in the total tumour-infiltrating T-lymphocyte may be low,^13^^,^^30^ it could be the case that tumour-infiltrating T-lymphocyte subtype analysis of our cohort might shed light on why we did not see a survival benefit similar to that reported in the literature. Furthermore, there exists a risk of publication bias, as noted by de Ruiter *et al*.,^11^ wherein statistically non-significant results for specific T-cell subtypes (e.g. CD4+) are not reported. Hence, while our results do not align with previous studies, it is important to publish these findings.

Interobserver reliability of tumour-infiltrating lymphocyte scoring is often reported as good to excellent,^12^^,^^20^ but this finding is not universal.^15^^,^^17^^,^^31^^,^^32^ De Keukeleire *et al*.^17^ reported moderate agreement (Fleiss’ *k* = 0.31–0.48) in interobserver tumour-infiltrating T-lymphocyte scores, as did Yin *et al*.^15^ Furthermore, tumour-infiltrating T-lymphocyte scoring from oropharyngeal specimens is ostensibly more challenging given the background lymphoid tissue.^15^^,^^33^ The use of artificial intelligence (AI)^32^^,^^34^^–^^36^ and machine learning methods^16^ to improve reliability and reproducibility of tumour-infiltrating T-lymphocyte scoring has been reported. Future studies or the adoption of tumour-infiltrating T-lymphocyte scoring in routine clinical practice may benefit from the assistance of AI and standardised methods.^33^

The variable nature of manual tumour-infiltrating T-lymphocyte scoring and inherent interobserver variability may be contributory factors to the proportion of cases categorised as low tumour-infiltrating T-lymphocytes in our cohort, which was 42.1 per cent. This is comparatively high with respect to other reports in which the low tumour-infiltrating T-lymphocytes proportion ranged from 9 to 34 per cent among patients with oropharyngeal SCC associated with HPV.^12^^,^^13^^,^^17^^,^^18^^,^^37^ While other clinicopathological characteristics may influence tumour-infiltrating T-lymphocyte density, there was no difference in terms of smoking (*p* = 0.61), N stage (*p* = 0.57) and overall stage (*p* = 0.26) between the high and low tumour-infiltrating T-lymphocytes groups in our study. Previous studies have similarly reported that tumour-infiltrating T-lymphocyte score is independent of smoking history.^13^^,^^37^ Almangush *et al*.^37^ found higher tumour-infiltrating T-lymphocyte density to be associated with lower T stage. This trend was observed in our cohort without reaching statistical significance (*p* = 0.17). The large proportion of low tumour-infiltrating T-lymphocytes cases in our cohort might have skewed survival outcomes.

Fifteen cases were excluded from our analysis because of lack of sufficient archived material. Other retrospective studies examining tumour-infiltrating T-lymphocytes in oropharyngeal SCC have had to discount cases for the same reason.^13^^,^^18^^,^^37^ This is, of course, a limitation of the retrospective design but calls into question the generalisability of results from incomplete biological datasets. Previous studies have identified the use of archived biological material as a potential source of bias.^38^^,^^39^ Thus, the use of archived material to inform the prognostic value of specific biomarkers should be approached with caution, with some authors suggesting adherence to certain criteria to do so.^40^ Insufficient archived material in 20 per cent of our cohort (15 of 74) may have influenced the outcomes observed.

Overexpression of p16 by more than 70 per cent is the accepted surrogate for HPV positivity in clinical practice.^4^^,^^41^ However, it is recognised that between 7 and 19 per cent of p16 positive cases are HPV DNA negative.^1^^,^^42^^–^^44^ HPV DNA polymerase chain reaction and/or in situ hybridisation was additionally conducted on 27 patients in this study cohort who showed HPV positivity concordant with p16 positivity. Of the 30 patients who had p16 testing only, there is a risk that these could, in fact, be HPV negative. That said, this risk is estimated to be low because of the high prevalence of oropharyngeal SCC associated with HPV in Ireland,^45^^,^^46^ and therefore unlikely to affect the overall results of this study.

In summary, five-year survival data did not differ based on tumour-infiltrating T-lymphocyte stratification in this study. This finding is in contrast to that in the published literature. Potential reasons for this are outlined above. Nevertheless, tumour-infiltrating T-lymphocyte scoring based on haematoxylin and eosin-stained slides, in general, shows promise of prognostic value in oropharyngeal SCC associated with HPV and may be particularly relevant as immunotherapy becomes more widely used in this disease. The findings of this study suggest further research is warranted. Prospective studies, adopting a standardised tumour-infiltrating T-lymphocyte assessment, should be undertaken to confirm its utility in oropharyngeal SCC associated with HPV prognosis before it can be implemented in routine clinical practice.

- Human papillomavirus (HPV)-associated squamous cell carcinoma (SCC) compared with non-HPV oropharyngeal SCC typically carries a better prognosis
- A subset of patients with oropharyngeal SCC associated with HPV experience poor outcomes
- Previous studies have suggested tumour-infiltrating lymphocytes can predict survival in oropharyngeal SCC associated with HPV
- The predictive value of tumour-infiltrating T-lymphocytes in oropharyngeal SCC associated with HPV is not upheld in this study
- Further evaluation of the prognostic utility of tumour-infiltrating T-lymphocytes in oropharyngeal SCC associated with HPV is required before it can be incorporated into routine practice

## Table 1 Long description

The table compares characteristics of patients with high versus low tumour-infiltrating T-lymphocytes. Patients with high infiltration are younger (mean age 56.5 years) compared to those with low infiltration (mean age 60.1 years), though the difference is not statistically significant (p=0.12). Both groups have similar follow-up durations and smoking histories. High infiltration is more common in males (87.9%) than females (12.1%), while low infiltration shows a slightly higher female percentage (20.8%). T stage distribution varies, with T4 more prevalent in low infiltration (33.3%) compared to high (9.1%). N stage and overall stage distributions show no significant differences between groups. The p values indicate no statistically significant differences across measured characteristics.

Navigate back to Table 1.

## Figure 1 Long description

The graph shows cumulative survival on the y-axis and time in months on the x-axis, ranging from 0 to 60 months. Two curves are plotted: one for high tumour-infiltrating T-lymphocytes (dashed line) and one for low tumour-infiltrating T-lymphocytes (solid line). The logrank p-value is 0.46, indicating no significant difference in survival between the groups. The high group shows slightly better survival, but both groups have similar outcomes over the period.

Navigate back to Figure 1.

## Figure 2 Long description

The graph shows cumulative survival on the y-axis and time in months on the x-axis, ranging from 0 to 60 months. Two curves are plotted: one for the high group (dashed line) and one for the low group (solid line). Both curves start at a cumulative survival of 1.00 and show a slight decline over time. The logrank p-value is 0.88, indicating no significant difference between the groups. A legend at the bottom identifies the line styles for high and low groups.

Navigate back to Figure 2.

## Figure 3 Long description

The graph shows cumulative survival on the y-axis and time in months on the x-axis, ranging from 0 to 60 months. Two curves are plotted: one for the high group (dashed line) and one for the low group (solid line). Both curves start at a cumulative survival of 1.00 and show a decline over time. The logrank p-value is 0.73, indicating the statistical comparison between the two groups. The legend identifies the dashed line as 'High' and the solid line as 'Low'.

Navigate back to Figure 3.
